# A new multiplex PCR for the accurate identification and differentiation of *Salmonella enterica* serovar Gallinarum biovars Pullorum and Gallinarum

**DOI:** 10.3389/fmicb.2022.983942

**Published:** 2022-09-06

**Authors:** Dan Xiong, Li Yuan, Li Song, Xinan Jiao, Zhiming Pan

**Affiliations:** ^1^Jiangsu Key Laboratory of Zoonosis, Yangzhou University, Yangzhou, Jiangsu, China; ^2^Jiangsu Co-innovation Center for Prevention and Control of Important Animal Infectious Diseases and Zoonoses, Yangzhou University, Yangzhou, Jiangsu, China; ^3^Key Laboratory of Prevention and Control of Biological Hazard Factors (Animal Origin) for Agrifood Safety and Quality, Ministry of Agriculture of China, Yangzhou University, Yangzhou, Jiangsu, China; ^4^Joint International Research Laboratory of Agriculture and Agri-product Safety of the Ministry of Education, Yangzhou University, Yangzhou, Jiangsu, China

**Keywords:** *Salmonella pullorum*, *Salmonella gallinarum*, multiplex PCR, *I137_14445*, *ybgL*, accurate differentiation

## Abstract

*Salmonella enterica* serovar Gallinarum biovars Gallinarum and Pullorum cause severe chicken salmonellosis, a disease associated with high mortality and morbidity among chickens worldwide. The conventional serotyping and biochemical reactions have been used to identify *Salmonella* serovars. However, the conventional methods are complicated, time-consuming, laborious, and expensive. Furthermore, it is challenging to distinguish *S.* Gallinarum and *S.* Pullorum *via* biochemical assays and serotyping because of their antigenic similarity. Although various PCR methods were established, a PCR protocol to detect and discriminate *S.* Gallinarum and *S.* Pullorum simultaneously is lacking. Herein, a one-step multiplex PCR method was established for the accurate identification and discrimination of *S.* Pullorum and *S.* Gallinarum. Three specific genes were used for the multiplex PCR method, with the *I137_14445* and *ybgL* genes being the key targets to identify and differentiate *S.* Gallinarum *and S.* Pullorum, and *stn* being included as a reference gene for the *Salmonella* genus. *In silico* analysis showed that the *I137_14445* gene is present in all *Salmonella* serovars, except for *S.* Gallinarum, and could therefore be used for the identification of *S.* Gallinarum. A 68-bp sequence deficiency in *ybgL* was found only in *S.* Pullorum compared to other *Salmonella* serovars, and this could therefore be used for the specific identification of *S.* Pullorum. The developed PCR assay was able to distinguish *S.* Gallinarum *and S.* Pullorum among 75 various *Salmonella* strains and 43 various non-*Salmonella* pathogens with excellent specificity. The detection limit for the genomic DNA of *S.* Gallinarum and *S.* Pullorum was 21.4 pg./μL, and the detectable limit for bacterial cells was 100 CFU. The developed PCR method was used for the analysis of *Salmonella* isolates in a chicken farm. This PCR system successfully discriminated *S.* Gallinarum *and S.* Pullorum from other different *Salmonella* serovars. The PCR results were confirmed by the conventional serotyping method. The newly established multiplex PCR is a simple, accurate, and cost-effective method for the timely identification and differentiation of *S.* Pullorum and *S.* Gallinarum.

## Introduction

*Salmonella enterica* can cause severe enteric fever, gastroenteritis, and septicemia, leading to serious public health problems globally (Foley and Lynne, 2008; Odoch et al., 2017; Williams et al., 2022). *Salmonella* spp. contains over 2,650 serovars by the diverse combinations of lipopolysaccharide (O antigens) and flagellar structure (H antigens; Guibourdenche et al., 2010; Cheng et al., 2019). However, some *Salmonella* serovars are only infectious to the specific hosts (Saeki et al., 2013; Zhu et al., 2015).

Fowl typhoid (FT) and Pullorum disease (PD) are caused by *Salmonella enterica* serovar Gallinarum biovars Gallinarum (*S. gallinarum*) and Pullorum (*S. pullorum*), respectively. The two types of septicemic diseases have significant effects on poultry industry, resulting in serious economic losses in many countries (Foley et al., 2011). PD, caused by *S. pullorum*, is associated with high mortality and morbidity rates among the young chicks, especially those less than 3 weeks of age. The characteristics of the infected chicks are the acute septicemia and white viscous diarrhea (Rettger, 1909). *S.* Pullorum can also be transmitted to the eggs through the ovary in the infected hens, resulting in both horizontal and vertical transmission and causing serious economic burden for the poultry industry (Geng et al., 2017; Xu et al., 2018). *S.* Gallinarum, a close relative of *S.* Pullorum, can lead to chronic and acute septicemia in different ages of poultry, and is a host-specific pathogen causing FT (Barrow and Freitas Neto, 2011).

The White–Kauffmann–Le Minor scheme has been used as the conventional method for *Salmonella* serotyping. The method is based on specific O (somatic) and H (flagellar) antisera by using slide agglutination tests (Majchrzak et al., 2014). However, this method is time-consuming, taking 5–6 days for the whole procedure. Besides, the expensive typing antisera are required for the assay (Bell et al., 2016). Furthermore, the differentiation of *S.* Pullorum and *S.* Gallinarum is difficult as the same O antigens 1, 9, and 12 between them (Christensen et al., 1993). Most *Salmonella* species possess flagella and exhibit motility. However, *S.* Pullorum *and S.* Gallinarum are two notable exceptions, having been shown lack of motility and flagella (H antigen; Holt and Chaubal, 1997). The biochemical tests, based on the fermentation types of ornithine, spironolactone, dulcitol, and maltose, were conducted previously to identify the two *Salmonella* biovars (Van Immerseel et al., 2013). However, the characteristics of typical and atypical colonies by the naked eyes increased the workload because the possibility of contamination must be excluded (Bai et al., 2019).

Molecular methods have shown high ability for the sensitive and specific discrimination of different pathogens and closely related variants. The increasing development of DNA-based detection methods has been applied to identify *Salmonella* spp., such as amplified fragment length polymorphism, pulsed-field gel electrophoresis, and DNA–DNA microarray hybridization (Swaminathan et al., 2001; Liebana, 2002; Morales et al., 2005). More recently, whole-genome sequencing has further enhanced the genetic analysis of *Salmonella*. The whole bacterial genome of each strain can be determined and the results can be compared to the large genetic databases (Carroll et al., 2021; Atlaw et al., 2022). However, this method is expensive and professional technicians are required for the data analysis.

Therefore, accurate and cost-effective methods for the fast and sensitive detection of specific *Salmonella* serovars in poultry products are urgently needed. A number of PCR methods have been developed, and the assays demonstrated high specificity and sensitivity in detecting *Salmonella* serotypes using such methods (Massi et al., 2003; Kim et al., 2017). The combination of PCR and restriction fragment length polymorphism was used for the differentiation of *S.* Pullorum *and S.* Gallinarum (Ribeiro et al., 2009; Soler-García et al., 2014). However, these complex analyses have limited the diagnostic applications of assays for the differential diagnosis of PD and FT (Ren et al., 2017). A rapid and cost-effective diagnostic method is therefore in urgent need to detect and differentiate *S.* Gallinarum *and S.* Pullorum.

In this study, a new one-step multiplex PCR method was developed for the specific detection and differentiation of *S.* Pullorum and *S.* Gallinarum simultaneously. The assay involved three pairs of primers based on the *I137_14445*, *ybgL*, and *stn* genes. The sensitivity and specificity of the multiplex PCR method were determined, and the assay was used for the specific identification of *S.* Gallinarum *and S.* Pullorum among clinical *Salmonella* isolates from a chicken farm.

## Materials and methods

### Bacterial strains

The bacterial strains including *Salmonella* spp. and non-*Salmonella* pathogens for the development and verification of the multiplex PCR method are listed in Supplementary Table S1. A total of 75 *Salmonella* strains and 43 various non-*Salmonella* strains were used in the present study, which were isolated previously as part of our routine monitoring and were stored at the Jiangsu Key Laboratory of Zoonosis, Yangzhou University. The biochemical identification of all *Salmonella* strains was conducted with the API identification kits (BioMérieux, Marcy, France). The *Salmonella* serovars were verified with the diagnostic antisera (Tianrun Bio-Pharmaceutical, Ningbo, China) in accordance with the White–Kauffmann–Le Minor scheme. For the discrimination of *S.* Pullorum and *S.* Gallinarum, the ornithine decarboxylation and dulcitol fermentation were conducted.

### Bacterial growth and genomic DNA extraction

Frozen stocks of the isolates were recovered on brain heart infusion (BHI) agar (Becton, Dickinson and Company, Sparks, MD, United States) or Luria–Bertani (LB) agar (Oxoid, Basingstoke, United Kingdom) overnight at 37°C. The bacterial strains were cultured in LB or BHI broth at 37°C for 16 h with 180 rpm for the genomic DNA extraction.

The extraction of bacterial genomic DNA was conducted with the TIANamp Bacteria DNA Kit (Tiangen, Beijing, China) in accordance with the manufacturer’s procedures. The DNA purity and concentration were determined with the NanoDrop ND-1000 (Thermo Scientific, Wilmington, DE, United States). The DNA was subsequently stored at –20°C prior to use.

### *In silico* analysis and primer design

To establish a useful PCR method to identify and differentiate the biovars *S.* Gallinarum and *S.* Pullorum, we analyzed differences in the nucleotide sequences of the *I137_14445* and *ybgL* genes. The genes *I137_14445* (GenBank acc. no. CP006575.1, region 3,087,481–3,088,224) and *ybgL* (GenBank acc. no. AM933173.1, region 770825-771559) were searched in the non-redundant nucleotide collection (nr/nt) database. The displayed number of nucleotide sequences was set to the maximum value of 5,000 to ensure that all aligned sequences in the database were included. DNA sequence alignment of *I137_14445* and *ybgL* genes from *S.* Pullorum, *S.* Gallinarum and other *Salmonella* serotypes was performed using ClustalW. Three pairs of primers were optimized for the development of the multiplex PCR. The positions of the designed primers were based on the gap in the *ybgL* gene, the unique sequence in the *I137_14445* gene, and a conserved sequence to all *Salmonella* serotypes in the *stn* gene. The primers were designed by length so that they could be distinguished on an agarose gel. The first primers of *I137_14445*-F/R amplified a 525-bp product to differentiate *S.* Gallinarum from other serovars. The second primer set, *ybgL*-F/R, amplified a 307-bp fragment to allow for the discrimination of *S.* Pullorum. The third primer set, *stn*-F/R, amplified a 731-bp fragment to identify the *Salmonella* genus from other microorganisms (Table 1). The three primer sets were designed and checked using the software Primer Premier 5.0. The primer specificity was determined with the basic local alignment search tool (BLASTn; NCBI, Bethesda, MD, United States). The primers were commercially synthesized by GenScript (Nanjing, China).

**Table 1:** Primer sequences for the specific detection and differentiation of *S. gallinarum* and *S. pullorum* with the multiplex PCR system.

Primers	Primer sequence (5′ → 3′)	Size (bp)	GenBank acc. no./Nt segments	*Salmonella* serovars		
				SP	SG	Non-SP/SG
*stn F*	TCCTGTTGTCTCGCTATCACTG	731	L16014.1 353–1,083	+	+	+
*stn R*	TTTTGGCATCAGCGTTATCAGC					
*I137_14445* F	AGCAGAAGAAGCCGTGTGAT	525	CP006575.13087691–3,088,215	+	−	+
*I137_14445* R	CCACGCCACAGTGATGTAGTAC					
*ybgL* F	AACCGCACGGTATGCTCTATAAC	307	AM933173.1771135–771,441	−	+	+
*ybgL* R	ACGCCAGTAACGCTTTTCACTC					

SP: *S. pullorum*; SG: *S. gallinarum*.

### Development and optimization of the multiplex PCR assay

The multiplex PCR system was conducted in a final volume of 25 μl, including 2 × Taq Master mix (12.5 μl; Vazyme, Nanjing, China), the *I137_14445* F/R primers (80 nM), the *ybgL*-F/R primers (80 nM), the *stn*-F/R primers (40 nM), and the bacterial genomic DNA (100 ng). The PCR reaction was conducted in a T100 Thermal Cycler (Bio-Rad, Hercules, CA, United States), and programmed as an initial denaturation at 94°C for 3 min; 30 sequential cycles of 94°C for 40 s, 53°C for 30 s, and 72°C for 60 s; and a final step of 72°C for 10 min. The PCR products were stained with GelRed Nucleic Acid Gel Stain (Biotium, Fremont, CA, United States) following 1% agarose gel electrophoresis. The visualization of the amplified PCR fragments was obtained by using the Gel Doc XR Gel Documentation System (Bio-Rad).

### Specificity of the multiplex PCR assay

The specificity of the multiplex PCR method was determined by using the genomic DNA extracted from 12 *S.* Pullorum strains, 4 *S.* Gallinarum strains, 59 strains from other *Salmonella* serovars, and 43 non-*Salmonella* pathogens. Detailed information for these strains is presented in Supplementary Table S1.

### Sensitivity of the multiplex PCR assay

The PCR sensitivity was conducted to evaluate the detection limit of the method. The bacterial genomic DNA was from the two *Salmonella* biovars *S.* Gallinarum strain SG9 and *S.* Pullorum strain S06004. A serial dilution (10-fold) was obtained from 21.4 to 2.14 pg./μl to serve as the templates in the developed multiplex PCR method. Overnight cultured bacteria of *S.* Pullorum and *S.* Gallinarum were washed twice with PBS and the optical densities at 600 nm (OD_600_) of the two strains were adjusted to 1. The CFU concentrations of the two strains when the OD_600_ = 1 were determined by plate counts. Freshly cultured bacteria were adjusted to the OD_600_ = 1 and diluted to the same CFU concentrations with PBS. The bacteria were serially diluted (10-fold) from 2 × 10^7^ to 2 × 10^3^ CFU/ml, and the bacterial concentrations were verified by plate counts. The genomic DNA was extracted from these diluted bacterial suspensions, and 5 μl of the extracted DNA was served as a template in the multiplex PCR.

### Analysis of chicken egg samples using the PCR assay

Naturally contaminated samples were collected from clinically dead eggs from a chicken farm in Yangzhou, China. The isolation of *Salmonella* pathogens from the chicken egg samples were conducted as previously described (Cai et al., 2016; Zhou et al., 2017). In brief, each sample was pre-enriched at 37°C for 24 h in 50 ml of buffered peptone water (Difco, BD, Sparks, MD, United States). The bacterial culture was streaked onto xylose lysine tergitol 4 (Difco, BD) agar, and incubated at 37°C for 16 h. The presumptive *Salmonella* colonies were individually picked as templates for the multiplex PCR. Meanwhile, each sample was also subjected to a standard traditional serum agglutination assay.

## Results and discussion

### Sequence alignment analysis and primer design

**Figure 1:**
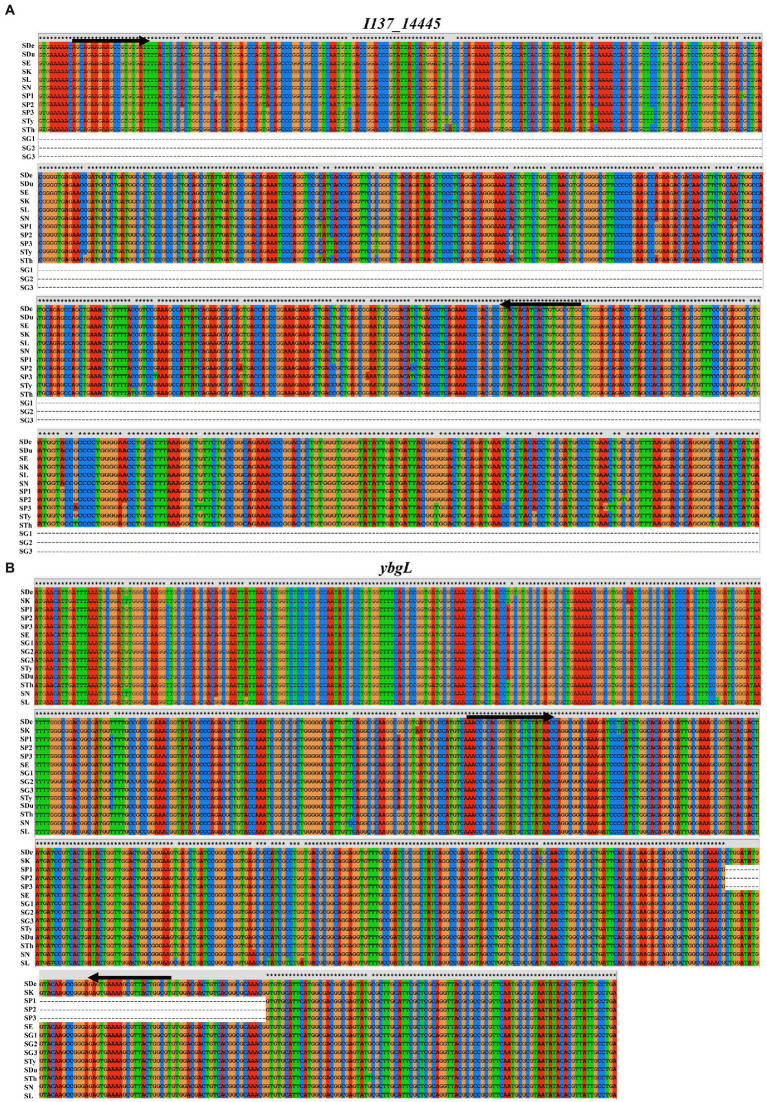
DNA sequence alignment of *I137_14445* and *ybgL* genes from *S.* Pullorum, *S.*Gallinarum, and other *Salmonella* serotypes. **(A)** The *I137_14445* gene is present in all *Salmonella* serovars except for *S.* Gallinarum, and this discrepancy could be used for the identification of *S.* Gallinarum. **(B)** A 68-bp region in the *ybgL* gene was present in *S.* Gallinarum and other *Salmonella* serovars but deleted in *S.* Pullorum, which allowed for the specific identification of *S.* Pullorum. The positions of the designed primers are indicated with the black arrows. SDe, *S. Derby* 2014LSAL02547 (GenBank acc. no. CP029486.1); SK, *S.* Kentucky 161,365 (GenBank acc. no. CP043664.1); SP1, *S.* Pullorum S06004 (GenBank acc. no. CP006575.1); SP2, *S.* Pullorum ATCC9120 (GenBank acc. no. CP012347.1); SP3, *S.* Pullorum CFSAN022627 (GenBank acc. no. CP075028.1); SE, *S.* Enteritidis P125109 (GenBank acc. no. CP063700.1); SG1, *S.* Gallinarum 287/91 (GenBank acc. no. AM933173.1); SG2, *S.* Gallinarum 9,184 (GenBank acc. no. CP019035.1); SG3, *S.* Gallinarum 07Q015 (GenBank acc. no. CP077760.1); STy, *S. typhimurium* FORC50 (GenBank acc. no. CP019383.1); SDu, S. Dublin CVM22429 (GenBank acc. no. CP032396.1); STh, *S.* Thompson SH11G0791 (GenBank acc. no. CP041171.1); SN, *S.* Newport CFSAN003387 (GenBank acc. no. CP016014.1); SL, *S.* London CVMN17S347 (GenBank acc. no. CP082711.1).

Due to the advantages of rapidity, specificity and sensitivity, PCR has been used widely to detect and identify the particular bacterial pathogens. The choice of target is key to the development of a specific and sensitive PCR assay. *In silico* analysis confirmed the presence of the *I137_14445* gene in all *Salmonella* serovars except for *S.* Gallinarum, indicating that this discrepancy could be used for the identification of *S.* Gallinarum. A 68-bp region in the *ybgL* gene was present in *S.* Gallinarum and other *Salmonella* serovars but deleted in *S.* Pullorum, and this region could therefore be targeted for the specific identification of *S.* Pullorum (Figure 1). Thus, the *I137_14445* and *ybgL* genes could be used as targets for the accurate identification and differentiation of *S.* Gallinarum *and S.* Pullorum, and the *stn* gene was used as a reference gene for the *Salmonella* genus. Specific primer sets were designed to target these three DNA fragments (Table 1).

Other new genes have been reported for the identification of *Salmonella*. The *ipaJ* gene of *S.* Pullorum was used for the development of a PCR method to detect *S.* Pullorum (Xu et al., 2018). Another PCR method was developed based on the *cigR* gene for the efficient detection of *Salmonella* and identification of *S.* Gallinarum*/*Pullorum (Zhou et al., 2020). However, these PCR methods could not identify and differentiate *S.* Pullorum *and S.* Gallinarum simultaneously. In this study, the *I137_14445* and *ybgL* genes were used as targets to differentiate *S.* Gallinarum *and S.* Pullorum for the first time.

### Specificity of the primers for *S.* Gallinarum *and S.* Pullorum identification and differentiation

The timely identification, genotyping, and serotyping of *Salmonella* pathogens could provide important information about the strain identification and source of infection during an outbreak (Gebreyes et al., 2006). Even so, most genotyping assays such as plasmid profile analysis, ribotyping, amplified fragment length polymorphism, and pulsed-field gel electrophoresis could not produce results consistent with *Salmonella* serotypes and genotypes (Bailey et al., 2002; Wang et al., 2011; Ozdemir and Acar, 2014).

**Figure 2:**
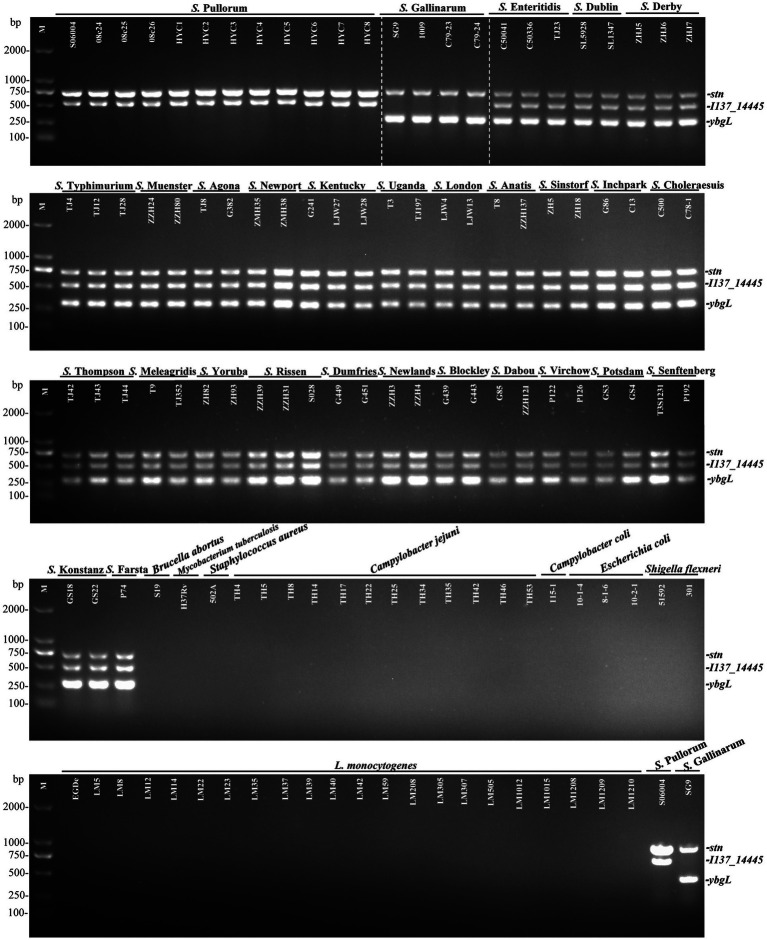
Specificity of the developed multiplex PCR to identify and differentiate *S.* Gallinarum and *S.* Pullorum. This developed PCR method was established on three specific targets: *stn* (731 bp), *I137_14445* (525 bp), and *ybgL* (307 bp). The PCR specificity was determined with extracted genomic DNA from 12 strains of *S.* Pullorum, 4 strains of *S.* Gallinarum, 59 strains of other *Salmonella* serovars, and 43 strains of non-*Salmonella* pathogens. Two specific PCR products for the *stn* and *I137_14445* genes could be amplified in *S.* Pullorum, while only products from the *stn* and *ybgL* targets could be generated in *S.* Gallinarum.

In this study, the specificity of the *I137_14445* and *ybgL* primer sets was evaluated with genomic DNA extracted from 75 *Salmonella* strains and 43 various non-*Salmonella* pathogens. The *Salmonella* strains included 29 various serovars (Supplementary Table S1). The results showed that for *S.* Pullorum, only two amplified products, 731-bp *stn* and 525-bp *I137_14445*, were generated. For *S.* Gallinarum, two products, 731-bp *stn* and 307-bp *ybgL*, were generated. For other *Salmonella* serovars, three products, *stn*, *I137_14445*, and *ybgL*, were amplified. By contrast, no bands were observed for the 43 non-*Salmonella* pathogens (Figure 2). No false negative or positive fragments were produced, suggesting that the developed multiplex PCR had high specificity. Both biovars could be identified and distinguished based on the newly developed multiplex PCR.

Previous methods were developed based on a single-nucleotide polymorphism or the variable regions of a certain gene to distinguish *S.* Gallinarum *and S.* Pullorum, such as PCR restriction fragment length polymorphism and single-stranded conformational polymorphism (Kisiela et al., 2005; Soler-García et al., 2014). However, in the present study, *S.* Gallinarum and *S.* Pullorum could be directly differentiated simultaneously based on three specific targets in a multiplex PCR. The multiplex PCR assay generated products of the *I137_14445* and *ybgL* genes, which enabled clear discrimination between *S.* Gallinarum*, S.* Pullorum, and other serovars. Importantly, as the primers *I137_14445* F/R are *S.* Gallinarum-specific, and the *ybgL* gene is *S.* Pullorum-specific, the two candidate genes could be used separately, to identify the two biovars, respectively.

### Sensitivity of the multiplex PCR assay for *S.* Gallinarum and *S.* Pullorum identification

**Figure 3:**
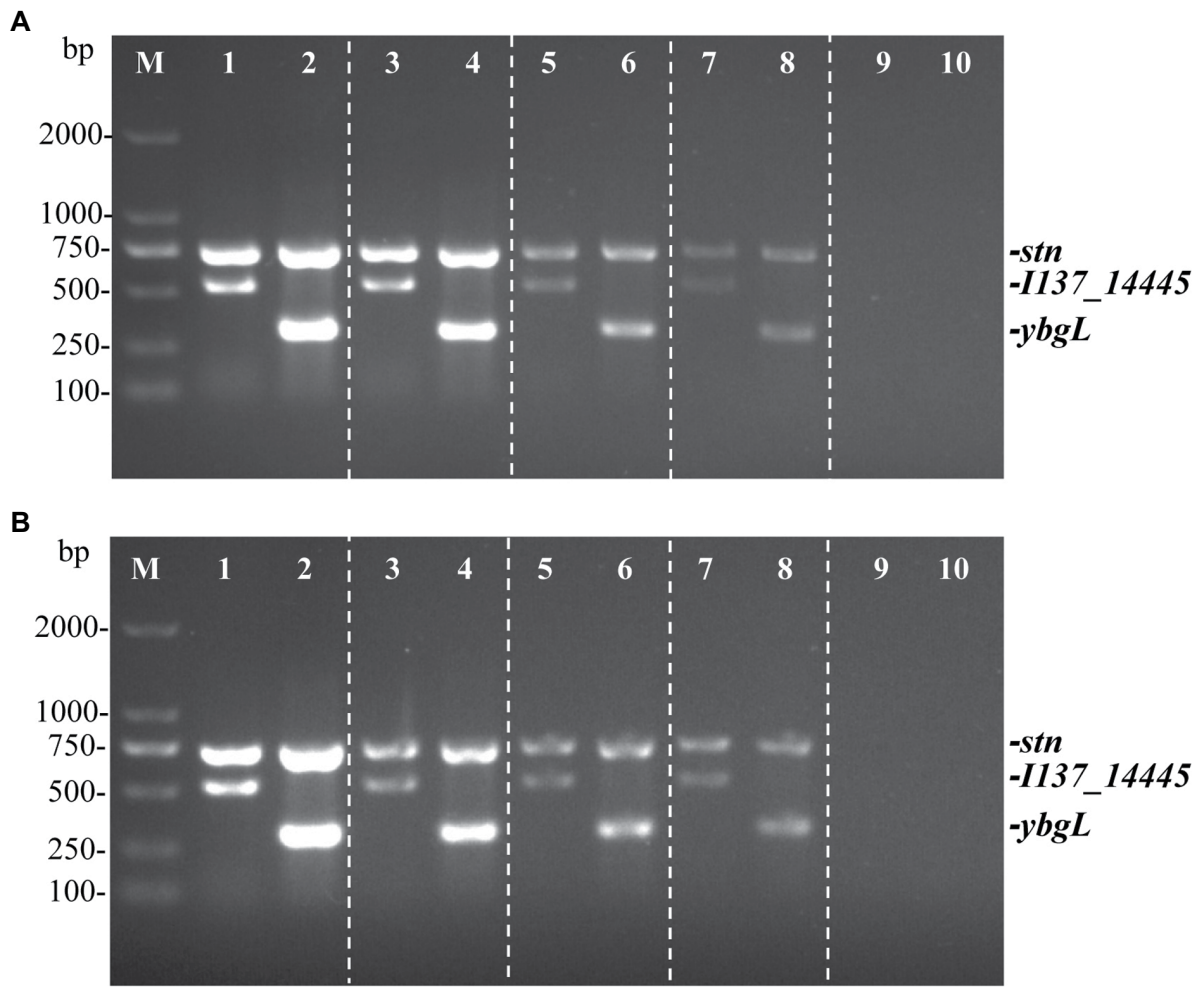
The multiplex PCR sensitivity to detect and differentiate *S.* Gallinarum (SG9) and *S.* Pullorum (S06004) with genomic DNA and cells. Three specific bands are amplified correlating with the *stn* (731 bp), *I137_14445* (525 bp), and *ybgL* (307 bp) genes. The sensitivity of the multiplex PCR was determined to detect the genomic DNA **(A)** and *Salmonella* cells **(B)**. Lanes 1, 3, 5, 7, 9 (*S.* Pullorum) and 2, 4, 6, 8, 10 (*S.* Gallinarum). Different concentrations of genomic DNA were used: 21.4 ng/μL, 2.14 ng/μL, 214 pg./μL, 21.4 pg./μL, 2.14 pg./μL; bacterial cells were used as template at the following concentrations: 10^5^, 10^4^, 10^3^, 10^2^, 10^1^ CFU.

The genomic DNA of *S.* Pullorum S06004 and *S.* Gallinarum SG9 was diluted serially from 21.4 ng/μl to 2.14 pg./μl to evaluate the detectable concentration of the PCR method. The minimum detection limit of the three sets of primers for the identification of *S.* Gallinarum *and S.* Pullorum was 21.4 pg./μl of genomic DNA (Figure 3A). The result was comparable to the HRM-PCR method established previously (Ren et al., 2017).

In addition, for different concentrations of bacterial cells, the PCR products were detected successfully at concentrations ranging from 10^5^ to 10^2^ CFU per reaction (Figure 3B). The minimum number of detectable cells of *S.* Gallinarum or *S.* Pullorum in the developed PCR method was much lower than that of the *sefA*-based PCR assay (400 CFU; Gong et al., 2016), and it was comparable to the *ipaJ*-based PCR assay (100 CFU; Xu et al., 2018). The results showed that the multiplex PCR method has low detection limit, and thereby low concentrations of *S.* Gallinarum *and S.* Pullorum could be detected. The lowest detection limit of each single PCR reaction was also determined. The results showed that the lowest number of cells of *S.* Pullorum *and S.* Gallinarum was 10 CFU for *ybgL* and *stn*, and 100 CFU for *I137_14445* (Supplementary Figure S1). As the detectable limit for bacterial cells of the developed multiplex PCR method was 100 CFU (Figure 3), the detectable limit of 100 CFU would be recommended for the accurate identification and differentiation of *S.* Pullorum *and S.* Gallinarum.

### Application of the *S.* Pullorum- and *S.* Gallinarum-specific multiplex PCR assay

*S.* Gallinarum *and S.* Pullorum can cause FT and PD respectively, and thus resulted in substantial economic losses of livestock (Ren et al., 2017). Some prevalent poultry bacterial diseases are caused by *S.* Gallinarum *and S.* Pullorum (Gong et al., 2014). Traditional assays for the identification of *Salmonella* serovars relied on diagnostic serological agglutination. However, the cross-reactivity could occur with *S.* Enteritidis or other serogroup D serovars (Ren et al., 2017).

The phenotypic characterization of *Salmonella* serovars has been mainly based on the serotyping. However, incorporation of serotyping analysis with other identification and molecular typing methods is often necessary for the rapid determination of the epidemiological linkage (Liu et al., 2011). Although various PCR methods have been developed to identify different serovars, including a multiplex qPCR assay (Rubio et al., 2017) and a duplex PCR assay (Batista et al., 2016) for detecting *S.* Gallinarum and *S.* Pullorum, few molecular methods are available to detect and differentiate *S.* Pullorum *and S.* Gallinarum simultaneously. The PCR assay developed in this study satisfies this requirement, and could contribute to the purification of the two *Salmonella* biovars in the poultry farms.

A total of 24 unknown serovars of *Salmonella* isolates were tested using our newly developed multiplex PCR method. The results showed that ten isolates produced the specific 731-bp target band of the *stn* gene and the 525-bp target band of the *I137_14445* gene, suggesting that these isolates were *S.* Pullorum. Two isolates produced the specific 731-bp target band of *stn* and the 307-bp target band of *ybgL*, indicating that these isolates were *S.* Gallinarum (Table 2). The multiplex PCR results were completely concordant with the conventional serotyping assay. Traditional serotyping method is complicated including non-selective and selective enrichment, biochemical identification, and serological characterization (Ren et al., 2017), which are time- and labor-intensive processes. The developed PCR assay presented in this study has the ability to detect and differentiate *S.* Pullorum and *S.* Gallinarum within 2 h, thereby greatly shortening the time required for serotype identification. However, it would not be able to identify the serovars if it is a mixture of *S.* Gallinarum/Pullorum and other *Salmonella* serotypes. A mixture of different *Salmonella* serovars will produce all three bands, and *S.* Pullorum and *S.* Gallinarum could not been identified and distinguished. Thus, the developed multiplex PCR method is suitable to identify the purified colonies or cultures.

**Table 2:** The developed multiplex PCR method was applied for the identification of *Salmonella* isolates from one chicken farm.

Serovar (no. of isolates)	Isolate no.	PCR results (*stn/I137_14445*/*ybgL*)	Dulcitol fermentation	Ornithine decarboxylase
Pullorum (10)	Ch4	+/+/−	−	+
	Ch5	+/+/−	−	+
	Ch9	+/+/−	−	+
	Ch10	+/+/−	−	+
	Ch11	+/+/−	−	+
	Ch12	+/+/−	−	+
	Ch16	+/+/−	−	+
	Ch17	+/+/−	−	+
	Ch18	+/+/−	−	+
	Ch24	+/+/−	−	+
Gallinarum (2)	Ch22	+/−/+	+	−
	Ch23	+/−/+	+	−
Enteritidis (9)	Ch1	+/+/+	/	/
	Ch2	+/+/+	/	/
	Ch3	+/+/+	/	/
	Ch6	+/+/+	/	/
	Ch7	+/+/+	/	/
	Ch8	+/+/+	/	/
	Ch13	+/+/+	/	/
	Ch14	+/+/+	/	/
	Ch15	+/+/+	/	/
Indiana (3)	Ch19	+/+/+	/	/
	Ch20	+/+/+	/	/
	Ch21	+/+/+	/	/

The traditional serotyping of the *Salmonella* isolates was determined following the White–Kauffmann–Le Minor scheme. The traditional differentiation of *Salmonella* biovars Pullorum and gallinarum was conducted with the ornithine decarboxylation and dulcitol fermentation.

## Conclusion

In summary, we established a multiplex PCR method that could detect and differentiate *S.* Pullorum *and S.* Gallinarum simultaneously based on three specific gene targets for the first time. Our assay exhibited efficient identification of both cultured bacteria and clinical *Salmonella* isolates. The results indicate that this multiplex PCR assay represents a one-step, economical, and accurate procedure for the sensitive, specific, and rapid identification of *S.* Gallinarum *and S.* Pullorum, respectively. The newly established PCR method could timely detect the presence of serovars Pullorum and Gallinarum accurately, especially when large quantities of samples to be tested, potentially facilitating the implementation of more timely and efficient control measures for PD or FT.

## Data availability statement

The original contributions presented in the study are included in the article/Supplementary material, further inquiries can be directed to the corresponding authors.

## Author contributions

XJ, ZP, and DX designed the experiments. DX, LY, and LS performed the PCR assays and analyzed the results. XJ, ZP, and DX wrote the paper. All authors contributed to the article and approved the submitted version.

## Funding

This work was supported by the National Natural Science Foundation of China (nos. 32102669, 31972685, and 31920103015), the Natural Science Foundation of Jiangsu Province (BK20210802), the China Postdoctoral Science Foundation (2020M681748), the Postdoctoral Science Foundation of Jiangsu Province (2021K095A), and the Priority Academic Program Development of Jiangsu Higher Education Institutions (PAPD).

## Conflict of interest

The authors declare that the research was conducted in the absence of any commercial or financial relationships that could be construed as a potential conflict of interest.

## Publisher’s note

All claims expressed in this article are solely those of the authors and do not necessarily represent those of their affiliated organizations, or those of the publisher, the editors and the reviewers. Any product that may be evaluated in this article, or claim that may be made by its manufacturer, is not guaranteed or endorsed by the publisher.

## Supplementary material

The Supplementary material for this article can be found online at: https://www.frontiersin.org/articles/10.3389/fmicb.2022.983942/full#supplementary-material
