# Supplementary material for: A new multiplex PCR for the accurate identification and differentiation of Salmonella enterica serovar Gallinarum biovars Pullorum and Gallinarum
Source: Front Microbiol. 2022 Sep 6;13:983942. doi: 10.3389/fmicb.2022.983942 (PMC9485580; doi:10.3389/fmicb.2022.983942)
Supplement: Supplementary file 1 [file Table_1.DOCX]

**Supplementary TABLE S1 | *Salmonella* and non-*Salmonella* strains used to evaluate the specificity and sensitivity of the developed multiplex PCR method.**

|  | Strain | Serovar/species | Source | Multiplex PCR results | | |
| --- | --- | --- | --- | --- | --- | --- |
|  |  |  |  | *stn* | *I137_14445* | *ybgL* |
| *Salmonella* | S06004 | Pullorum | Laboratory stock | + | + | – |
|  | 08c24 | Pullorum | Isolate from chicken | + | + | – |
|  | 08c25 | Pullorum | Isolate from chicken | + | + | – |
|  | 08c26 | Pullorum | Isolate from chicken | + | + | – |
|  | HYC1 | Pullorum | Laboratory stock | + | + | – |
|  | HYC2 | Pullorum | Laboratory stock | + | + | – |
|  | HYC3 | Pullorum | Laboratory stock | + | + | – |
|  | HYC4 | Pullorum | Laboratory stock | + | + | – |
|  | HYC5 | Pullorum | Laboratory stock | + | + | – |
|  | HYC6 | Pullorum | Laboratory stock | + | + | – |
|  | HYC7 | Pullorum | Laboratory stock | + | + | – |
|  | HYC8 | Pullorum | Laboratory stock | + | + | – |
|  | SG9 | Gallinarum | Laboratory stock | + | – | + |
|  | 1009 | Gallinarum | Laboratory stock | + | – | + |
|  | C79-23 | Gallinarum | Laboratory stock | + | – | + |
|  | C79-24 | Gallinarum | Laboratory stock | + | – | + |
|  | C50041 | Enteritidis | Laboratory stock | + | + | + |
|  | C50336 | Enteritidis | Laboratory stock | + | + | + |
|  | TJ23 | Enteritidis | Cai et al., 2016 | + | + | + |
|  | SL5928 | Dublin | Laboratory stock | + | + | + |
|  | SL1347 | Dublin | Laboratory stock | + | + | + |
|  | ZHJ5 | Derby | Isolate from pig | + | + | + |
|  | ZHJ6 | Derby | Isolate from pig | + | + | + |
|  | ZHJ7 | Derby | Isolate from pig | + | + | + |
|  | TJ4 | Typhimurium | Cai et al., 2016 | + | + | + |
|  | TJ12 | Typhimurium | Cai et al., 2016 | + | + | + |
|  | TJ28 | Typhimurium | Cai et al., 2016 | + | + | + |
|  | ZZH24 | Muenster | Zhou et al., 2017 | + | + | + |
|  | ZZH80 | Muenster | Zhou et al., 2017 | + | + | + |
|  | TJ8 | Agona | Cai et al., 2016 | + | + | + |
|  | G382 | Agona | Laboratory stock | + | + | + |
|  | ZMH35 | Newport | Laboratory stock | + | + | + |
|  | ZMH38 | Newport | Laboratory stock | + | + | + |
|  | G241 | Kentucky | Laboratory stock | + | + | + |
|  | LJW27 | Kentucky | Cai et al., 2016 | + | + | + |
|  | LJW28 | Kentucky | Cai et al., 2016 | + | + | + |
|  | T3 | Uganda | Cai et al., 2016 | + | + | + |
|  | TJ197 | Uganda | Cai et al., 2016 | + | + | + |
|  | LJW4 | London | Cai et al., 2016 | + | + | + |
|  | LJW13 | London | Cai et al., 2016 | + | + | + |
|  | T8 | Anatis | Laboratory stock | + | + | + |
|  | ZZH137 | Anatis | Zhou et al., 2017 | + | + | + |
|  | ZH5 | Sinstorf | Zhou et al., 2017 | + | + | + |
|  | ZH18 | Sinstorf | Zhou et al., 2017 | + | + | + |
|  | G86 | Inchpark | Laboratory stock | + | + | + |
|  | C13 | Inchpark | Laboratory stock | + | + | + |
|  | C500 | Choleraesuis | Laboratory stock | + | + | + |
|  | C78-1 | Choleraesuis | Laboratory stock | + | + | + |
|  | TJ42 | Thompson | Cai et al., 2016 | + | + | + |
|  | TJ43 | Thompson | Cai et al., 2016 | + | + | + |
|  | TJ44 | Thompson | Cai et al., 2016 | + | + | + |
|  | T9 | Meleagridis | Laboratory stock | + | + | + |
|  | TJ352 | Meleagridis | Cai et al., 2016 | + | + | + |
|  | ZH82 | Yoruba | Zhou et al., 2017 | + | + | + |
|  | ZH93 | Yoruba | Zhou et al., 2017 | + | + | + |
|  | ZZH39 | Rissen | Zhou et al., 2017 | + | + | + |
|  | ZZH31 | Rissen | Zhou et al., 2017 | + | + | + |
|  | S028 | Rissen | Isolate from pig | + | + | + |
|  | G449 | Dumfries | Laboratory stock | + | + | + |
|  | G451 | Dumfries | Laboratory stock | + | + | + |
|  | ZZH3 | Newlands | Zhou et al., 2017 | + | + | + |
|  | ZZH4 | Newlands | Zhou et al., 2017 | + | + | + |
|  | G439 | Blockley | Laboratory stock | + | + | + |
|  | G443 | Blockley | Laboratory stock | + | + | + |
|  | G85 | Dabou | Laboratory stock | + | + | + |
|  | ZZH121 | Dabou | Zhou et al., 2017 | + | + | + |
|  | P122 | Virchow | Laboratory stock | + | + | + |
|  | P126 | Virchow | Laboratory stock | + | + | + |
|  | GS3 | Potsdam | Isolate from duck | + | + | + |
|  | GS4 | Potsdam | Isolate from duck | + | + | + |
|  | T3S1231 | Senftenberg | Laboratory stock | + | + | + |
|  | P192 | Senftenberg | Laboratory stock | + | + | + |
|  | GS18 | Konstanz | Laboratory stock | + | + | + |
|  | GS22 | Konstanz | Laboratory stock | + | + | + |
|  | P74 | Farsta | Laboratory stock | + | + | + |
| Non-*Salmonella* | S19 | *Brucella abortus* | Laboratory stock | – | – | – |
|  | H37Rv | *Mycobacterium tuberculosis* | ATCC 27294 | – | – | – |
|  | 502A | *Staphylococcus aureus* | ATCC 27217 | – | – | – |
|  | TH4 | *Campylobacter jejuni* | Laboratory stock | – | – | – |
|  | TH5 | *Campylobacter jejuni* | Laboratory stock | – | – | – |
|  | TH8 | *Campylobacter jejuni* | Laboratory stock | – | – | – |
|  | TH14 | *Campylobacter jejuni* | Laboratory stock | – | – | – |
|  | TH17 | *Campylobacter jejuni* | Laboratory stock | – | – | – |
|  | TH22 | *Campylobacter jejuni* | Laboratory stock | – | – | – |
|  | TH25 | *Campylobacter jejuni* | Laboratory stock | – | – | – |
|  | TH34 | *Campylobacter jejuni* | Laboratory stock | – | – | – |
|  | TH35 | *Campylobacter jejuni* | Laboratory stock | – | – | – |
|  | TH42 | *Campylobacter jejuni* | Laboratory stock | – | – | – |
|  | TH46 | *Campylobacter jejuni* | Laboratory stock | – | – | – |
|  | TH53 | *Campylobacter jejuni* | Laboratory stock | – | – | – |
|  | 115-1 | *Campylobacter coli* | Isolate from chicken | – | – | – |
|  | 10-1-4 | *Escherichia coli* | Isolate from pig | – | – | – |
|  | 8-1-6 | *Escherichia coli* | Isolate from pig | – | – | – |
|  | 10-2-1 | *Escherichia coli* | Isolate from pig | – | – | – |
|  | 51592 | *Shigella flexneri* | Laboratory stock | – | – | – |
|  | 301 | *Shigella flexneri* | Laboratory stock | – | – | – |
|  | EGDe | *Listeria monocytogenes* | ATCC BAA-679 | – | – | – |
|  | LM5 | *Listeria monocytogenes* | Isolate from chicken | – | – | – |
|  | LM8 | *Listeria monocytogenes* | Isolate from chicken | – | – | – |
|  | LM12 | *Listeria monocytogenes* | Isolate from chicken | – | – | – |
|  | LM14 | *Listeria monocytogenes* | Isolate from chicken | – | – | – |
|  | LM22 | *Listeria monocytogenes* | Isolate from chicken | – | – | – |
|  | LM23 | *Listeria monocytogenes* | Isolate from chicken | – | – | – |
|  | LM35 | *Listeria monocytogenes* | Isolate from chicken | – | – | – |
|  | LM37 | *Listeria monocytogenes* | Isolate from chicken | – | – | – |
|  | LM39 | *Listeria monocytogenes* | Isolate from chicken | – | – | – |
|  | LM40 | *Listeria monocytogenes* | Isolate from chicken | – | – | – |
|  | LM42 | *Listeria monocytogenes* | Isolate from chicken | – | – | – |
|  | LM59 | *Listeria monocytogenes* | Isolate from chicken | – | – | – |
|  | LM208 | *Listeria monocytogenes* | Isolate from chicken | – | – | – |
|  | LM305 | *Listeria monocytogenes* | Isolate from chicken | – | – | – |
|  | LM307 | *Listeria monocytogenes* | Isolate from chicken | – | – | – |
|  | LM505 | *Listeria monocytogenes* | Isolate from chicken | – | – | – |
|  | LM1012 | *Listeria monocytogenes* | Isolate from chicken | – | – | – |
|  | LM1015 | *Listeria monocytogenes* | Isolate from chicken | – | – | – |
|  | LM1208 | *Listeria monocytogenes* | Isolate from chicken | – | – | – |
|  | LM1209 | *Listeria monocytogenes* | Isolate from chicken | – | – | – |
|  | LM1210 | *Listeria monocytogenes* | Isolate from chicken | – | – | – |
