# Supplementary material for: A new multiplex PCR for the accurate identification and differentiation of Salmonella enterica serovar Gallinarum biovars Pullorum and Gallinarum
Source: Front Microbiol. 2022 Sep 6;13:983942. doi: 10.3389/fmicb.2022.983942 (PMC9485580; doi:10.3389/fmicb.2022.983942)
Supplement: Supplementary file 2 [file Data_Sheet_1.PDF]

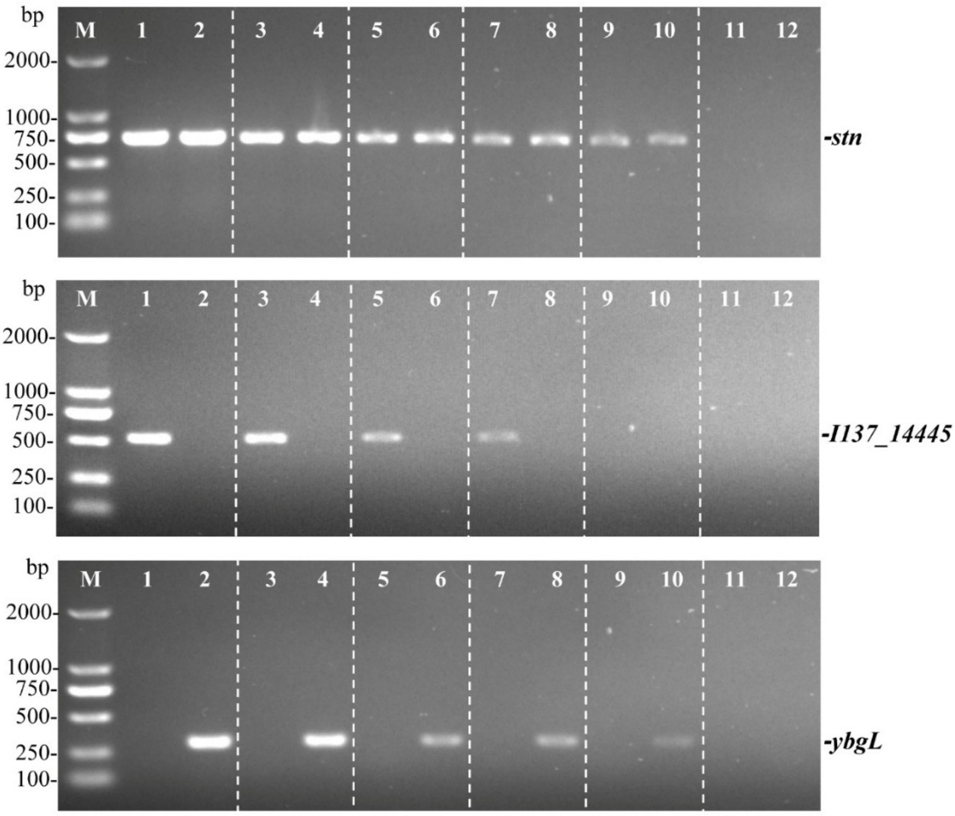

Supplementary FIGURE S1 | The sensitivity of the single PCR reaction was determined to detect *S.* Pullorum (S06004) and *S.* Gallinarum (SG9). Three specific bands are amplified respectively, correlating with the *stn* (731 bp), *I137_14445* (525 bp), and *ybgL* (307 bp). Lanes 1, 3, 5, 7, 9, 11 (*S.* Pullorum) and 2, 4, 6, 8, 10, 12 (*S.* Gallinarum). Bacterial cells were used as template at the following concentrations: 10^5, 10^4, 10^3, 10^2, 10^1, 10^0 CFU.
